# Comparative analysis of intraoral scanners accuracy using 3D software: an in vivo study

**DOI:** 10.1186/s40510-022-00416-5

**Published:** 2022-07-04

**Authors:** Federica Pellitteri, Paolo Albertini, Angelica Vogrig, Giorgio Alfredo Spedicato, Giuseppe Siciliani, Luca Lombardo

**Affiliations:** 1 Department of Orthodontics, University of Ferrara, Via Luigi Borsari, 46, 44121 Ferrara, Italy; 2 Faculty of Banking and Insurance, Catholic University of Milan, Largo Agostino Gemelli 1, 20123 Milan, Italy; 3 School of Dentistry, University of Ferrara, Via Luigi Borsari 46, 44121 Ferrara, Italy; 4 School of Orthodontics, University of Ferrara, Via Luigi Borsari 46, 44121 Ferrara, Italy

**Keywords:** Intraoral scanners, Accuracy, 3D systems

## Abstract

**Background:**

The purpose of the present in vivo study was to compare the accuracy, in terms of trueness, between full-arch digital impressions of different intraoral scanning systems, using as a reference the ideality of the conventional impression technique.

**Methods:**

Polyvinyl siloxane (PVS) two-step technique impressions of 27 subjects were taken, and the stone casts were scanned using desktop scanners R500 3Shape. For each arch, in vivo scans were taken with intraoral scanners Carestream CS3600, CEREC Omnicam and Trios 3Shape. All the files were compared, superimposing them on the reference model to calculate the total 3D and 2D deviations. The efficiency of the digital and conventional workflows was evaluated by measuring the work time in minutes. Statistical analyses were performed using R software (R Core Team 2020) with a *p*-value < 0.05.

**Results:**

The three intraoral scanners differed from the PVS impression by differences of the order of 100–200 µm, and there was a trend of greater imprecision in the molar area in both dental arches. In comparison with PVS technique, CEREC tended to reduce the size of the impression, Trios presented the trend of greater precision, and Carestream showed minor differences the transversal distance. The areas of greatest discrepancy both in excess and in defect with respect to the PVS impression were the molar areas and incisal margins. Trios 3Shape recorded the shortest times and therefore with a more performing speed.

**Conclusion:**

The Trios 3Shape was found to be the most accurate single-tooth scanner, while the Carestream CS 3600 showed better inter-arch diameter performance compared to PVS impressions. The 3D and 2D analyses showed a trend of greater distortion of the impressions compared to the conventional one in the molar region.

## Background

The information required to diagnose a malocclusion and develop an orthodontic treatment plan is provided by study models, photographs, radiographs and clinical examination [1]. The current gold standard is the conventional impression, made with an elastomeric material or custom trays [2].

From 1973, a new concept of oral impression was introduced, employing an intraoral scanning system. A few years later, a chair-side scanning device utilizing CAD/CAM technology was available commercially and manufactured by Sirona Dental Systems (CEREC) [2].

Intraoral scanners (IOS) are powerful devices used for optical impressions and are able to collect information on the shape and size of the dental arches through the emission of a light beam [3, 4]. The information collected by high-resolution cameras and processed by powerful software that derives from the genesis of a "cloud of points" a polygonal mesh, representing the scanned object; the scan is further processed to obtain the final 3D model [4, 5]. All intraoral scanners work with noninvasive optical technologies, as they do not have contact with the scanned object, such as confocal microscopy, light triangulation, and the active sampling wavefront. Each device uses more than one of these technologies to minimize noise from intraoral scanning, eliminate distortions due to the presence of saliva by standardizing the surfaces to be scanned with different optical properties and maximize the result. [6]

These .stl files allow to reduce the storage space, to improve and accelerate the communication with technicians or colleagues and to eliminate the discomfort of the traditional impressions; however, the lack of scientific literature does not clarify how much the accuracy of intraoral scanners approaches that of traditional silicones impressions.

Optical impressions could be used as a starting point for the creation of virtual setups aimed at creating a whole series of customized orthodontic devices. The most accurate orthodontic appliances, as expanders, mini-screw assisted devices, aligners or lingual orthodontics, require a construction precision in order to create individualized appliances for each patient, which can be efficient and effective in solving the malocclusion. [6–13].

In vitro studies regarding intraoral scanners report satisfactory results [8, 14, 15] but only through the comparison of different techniques used in vivo, is possible to understand the limits and advantages of these systems in the orthodontic field.

For these reasons, the purpose of the present in vivo study was to compare the accuracy in terms of trueness, between full-arch digital impressions of different intraoral scanning systems, using as a reference the ideality of the conventional impression technique.

The null hypothesis was that there would be no statistically significant differences in mean of trueness between the different digital impression system, and no statistically significant differences between them and the conventional impression technique.

## Methods

After the ethical approval of the institutional review board of the Postgraduate School of Orthodontics of the University of Ferrara and the informed consent release, twenty-seven consecutive subjects were included the study from the September 14 until the October 14, 2021: 11 males and 16 females between 15 and 29 years of age. Inclusion criteria were full natural permanent dentition (excluding third molars extracted or not erupted), the absence of amalgam or prosthetic restorations, the absence of orthodontic appliance.

First, polyvinyl siloxane two-step technique impressions were taken (A.V.) and then poured in stone casts (type IV). Subsequently, the stone casts were scanned using desktop scanners R500 3Shape (3Shape, Copenhagen, Denmark). Afterward, three intraoral scanners were used to scan the subjects’ full arch dentitions by the same operator: Carestream CS3600 (3.1, CS 3600®, Carestream, Rochester, NY, USA), CEREC Omnicam (Software 4.6.1, Cerec Omnicam®, Dentsply Sirona, Germany), Trios 3Shape (Software 1.18.2.6, Trios 3®, 3-Shape, Copenhagen, Denmark). All scans were recorded with the most accurate sequence of scans described in the literature: starting at the occlusal-palatal surfaces of the maxillary right second molar, moving toward the other side of the arch and always including two surfaces, and returning from the buccal side [16]. All the digital and conventional impressions were taken by the same expert clinician the same day, avoiding soft tissue fluctuations. A total of 216 files were obtained (27 subjects with 2 dental arches each, undergone to 4 impression techniques).

The efficiency of the digital and conventional workflows was evaluated by measuring the work time in minutes. For the conventional impressions were summed the manufacturer’s provided working times for the adhesive, impression material, antagonist impression material. For IOS, the effective work time was calculated as the sum of the addictive scan time, the software’s refinement process and the .stl files exportation.

As the literature reported, to measure scans accuracy, the data collected (.stl files) of both complete arch of each patient were imported into surface-matching software Geomagic X Control (3D Systems Inc, Rock Hill, SC) [17–19]. Each scan was superimposed with the scanned stone cast (162 superimpositions) using first the software’s initial matching algorithm tool (A.V.) and then the best fit alignment. The repeatability of the measurements was verified.

Measurements taken on the scanned stone cast were considered the reference unit, on which 7 points were inserted for each dental element (A.V.) from the second molar to the canine, of the upper and lower arches: most gingival point of FACC (Facial Axis of the Clinic Crown), point FA (Facial Axis point), most occlusal point of FACC, deepest point of the sulcus on the occlusal surface, most occlusal point of LACC (Lingual Axis of the Clinic Crown), point LA (Lingual Axis point), most gingival point of LACC [15, 20]. In order to verify the accuracy in its meaning of trueness of the three digital systems compared to the conventional technique, the next step was to perform again, after 14 days, the same measurements in the data of reference for all 27 subjects by a second operator (F.P.). This is to verify the intra-operator repeatability of the measurements and therefore that any discrepancies between the same points inserted in two different files did not depend on the operator. All these distances were compared to detect any statistically significant differences, such as to assert that the distances measured between the points of the reference data and the same points of the three measured data did not depend exclusively on the positioning of the points and therefore on the operator, but also and above all by the distortion (trueness error) of the scanner used. Moreover, the arches transverse dimensions and the distances between the same points of single tooth were assessed.

**Fig. 1:**
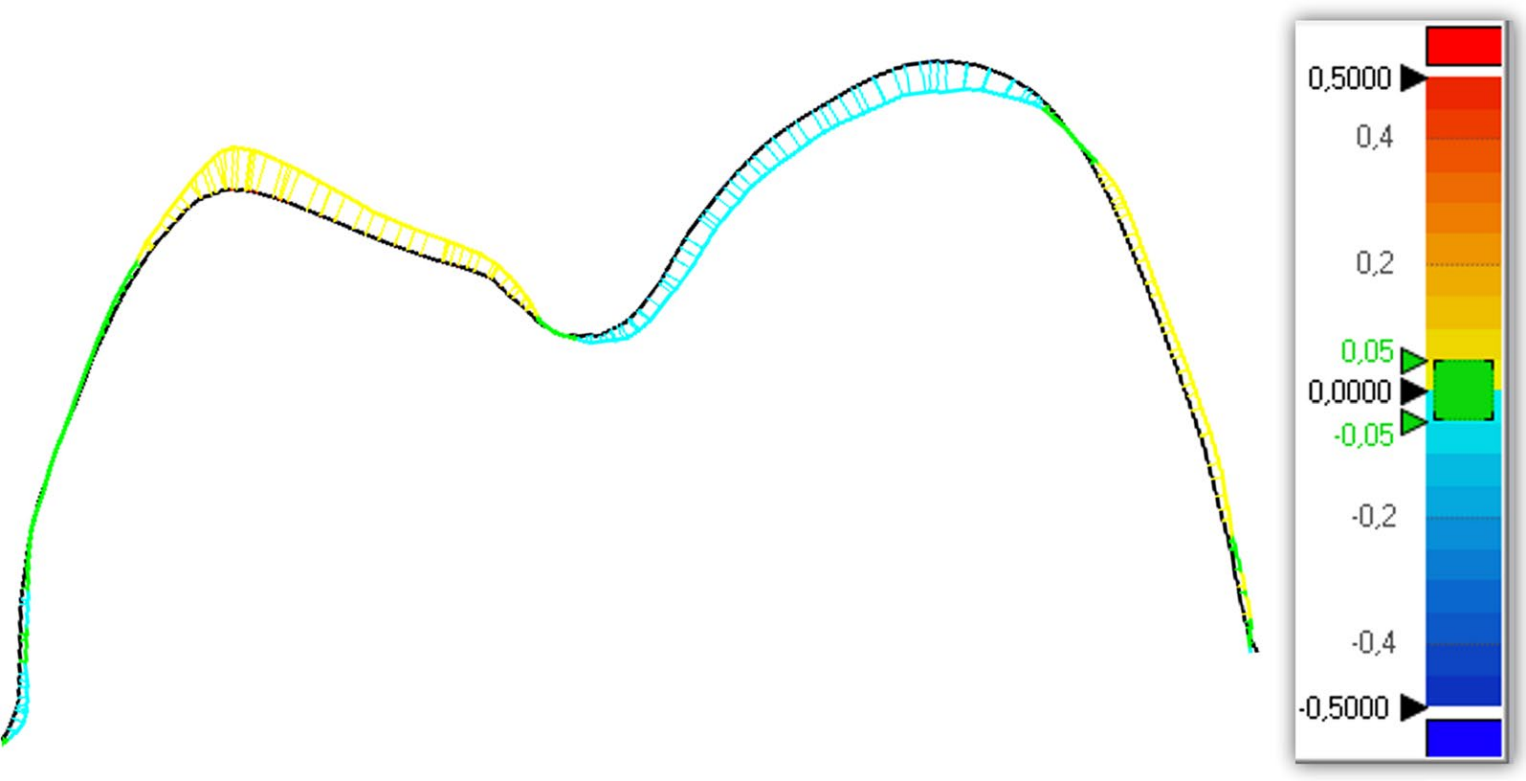
Cross section of the color-coded superimposed image

Subsequently, all the files were compared, superimposing them on the reference models to calculate the total 3D and 2D deviations (X, Y, and Z) between the data sets obtained from the reference scanner and the different intraoral scanners included in the study. Geomagic X Control allows detection automatically of discrepancies in micrometers thanks to the “3D comparison” tool, both positive (expansion) and negative (contraction). Deviations are viewed on a color-coded superimposed image considering the discrepancy range from 0.5 to 0 mm and from 0 to −0.5 mm highly reproducible [17] (Fig. 1).

### Statistical analysis

A dataset containing 3780 pairs of points measured on the files and 270 pairs of interarch distances concerning the PVS impressions of the 27 subjects in the sample and related Euclidean distances was collected (A.V., F.P.); similarly, the statistical analysis aims to evaluate the repeatability of distance measurements.

Descriptive statistics (mean, median, standard deviation and coefficient of variation) for linear measurements and intra-arch distances has been described.

In a second analysis, the differences between both the linear measurements distances on unit A (PVS impression) will be compared with respect to unit A (repeatability) and units B (Carestream CS 3600), C (CEREC Omnicam) and D (Trios 3Shape) extended to the study sample (27 subjects), to analyze reproducibility.

Subsequently, the differences between units B, C and D will be compared (comparison B–C, B–D, C–D) both for linear measurements and for interarch distances.

Finally, a *post hoc* analysis has been performed in order to verify, for each tooth, for which units the linear distances were statistically significant.

Statistical analyses were performed using R software (R Core Team 2020) with a *p*-value < 0.05 [21]. Descriptive statistic was used to analyze the results of chair time.

## Results

Tables 1 and 2 show the descriptive statistics (mean, median, standard deviation and coefficient of variation) for linear measurements and intra-arch distances. The mean of the distance indicates the mean imprecision, while the measures of variability indicate how much this imprecision is variable.

**Table 1:** Descriptive analysis on the average imprecision of linear distances

	Mean	Median	SD	Cv	Significance
*Upper arch*					
13	0.145	0.114	0.148	1.023	*
23	0.136	0.114	0.119	0.878	*
14	0.132	0.108	0.137	1.035	*
24	0.136	0.118	0.119	0.879	*
15	0.141	0.117	0.137	0.975	*
25	0.132	0.114	0.115	0.73	*
16	0.160	0.125	0.163	1.017	*
26	0.153	0.129	0.139	0.913	*
17	0.209	0.157	0.252	1.207	*
27	0.172	0.139	0.176	1.026	*
*Lower arch*					
33	0.173	0.137	0.179	1.039	*
43	0.157	0.128	0.144	0.915	*
34	0.149	0.137	0.116	0.774	*
44	0.144	0.118	0.127	0.882	*
35	0.160	0.139	0.136	0.854	*
45	0.157	0.139	0.129	0.823	*
36	0.180	0.145	0.158	0.878	*
37	0.243	0.186	0.260	1.067	*

SD: standard deviation

Cv: variation coefficient

*p < .0001

**Table 2:** Descriptive analysis on the average imprecision of intra-arch distances

	Mean	Median	Sd	Cv	Significance
*Upper arch*					
13–23	−0.060	−0.003	0.220	−3.64	*
14–24	0.022	0.011	0.188	8.67	*
15–25	0.023	0.007	0.141	6.19	*
16–26	−0.002	0.000	0.139	−56.62	*
17–27	−0.061	−0.001	0.276	−4.53	*
*Lower arch*					
43–33	−0.007	0.005	0.199	−29.98	*
44–34	0.025	0.010	0.155	6.22	*
45–35	0.055	0.024	0.183	3.31	*
46–36	−0.056	−0.001	0.283	−5.05	*
47–37	−0.006	0.003	0.304	−48.75	*

SD standard deviation

Cv: variation coefficient

*p < .0001

Table 3 shows the repeatability of linear measurements (mm) of STL files related to PVS impression. The reproducibility on unit PVS impression (A) compared to Carestream CS 3600 (B), CEREC Omnicam (C) and Trios 3Shape (D) was carried out on the 27 subjects in the sample (Table 4) and highlighted a trend of lower precision in linear measurements distances carried out at the level of the posterior sectors, in particular in the molar area. The three intraoral scanners differed from the PVS impression by differences of the order of 100–200 µm, and there was a trend of greater imprecision in the molar area in both dental arches, highlighting a statistically significant difference (p < 0.05) in the linear measurements made between PVS impression and Carestream, CEREC, Trios.

**Table 3:** Repeatability of linear measurements (mm) of STL files related to PVS impression

	Mean	Median	SD	Cv	Significance
*Upper arch*					
13	0.019	0.007	0.041	2.141	*
23	0.025	0.025	0.058	2.325	*
14	0.021	0.008	0.052	2.447	*
24	0.018	0.018	0.008	2.239	*
15	0.023	0.023	0.085	3.689	*
25	0.018	0.018	0.036	2.010	*
16	0.020	0.020	0.120	2.699	*
26	0.021	0.021	0.118	1.351	*
17	0.027	0.027	0.092	2.234	*
27	0.021	0.021	0.091	1.691	*
*Lower arch*					
33	0.060	0.015	0.055	2.007	*
43	0.068	0.018	0.029	1.732	*
34	0.058	0.020	0.060	1.572	*
44	0.054	0.016	0.036	1.696	*
35	0.062	0.019	0.100	1.616	*
45	0.057	0.016	0.094	1.663	*
36	0.053	0.019	0.080	1.507	*
46	0.061	0.015	0.100	1.640	*
37	0.054	0.054	0.090	1.673	*
47	0.056	0.017	0.092	1.639	*

SD: standard deviation

Cv: variation coefficient

*p < .0001

**Table 4:** Reproducibility of linear measurements (mm) of STL files between PVS impression (unit A) and unit B (Carestream CS 3600), unit C (CEREC Omnicam) and unit D (Trios 3Shape)

	Unit	Mean	Median	Std	Cv	Significance
*Upper arch*						
13	B	0.205	0.169	0.167	0.811	*
	C	0.182	0.142	0.156	0.855	*
	D	0.174	0.139	0.114	0.659	*
23	B	0.178	0.161	0.100	0.563	*
	C	0.172	0.145	0.120	0.697	*
	D	0.170	0.146	0.114	0.671	*
14	B	0.176	0.152	0.138	0.786	*
	C	0.177	0.133	0.155	0.878	*
	D	0.155	0.126	0.112	0.723	*
24	B	0.192	0.178	0.108	0.562	*
	C	0.166	0.135	0.116	0.700	*
	D	0.167	0.140	0.107	0.641	*
15	B	0.205	0.173	0.145	0.709	*
	C	0.166	0.142	0.120	0.722	*
	D	0.169	0.141	0.115	0.684	*
25	B	0.179	0.152	0.102	0.572	*
	C	0.165	0.131	0.118	0.715	*
	D	0.166	0.151	0.100	0.602	*
16	B	0.215	0.173	0.165	0.770	*
	C	0.211	0.165	0.165	0.770	*
	D	0.195	0.155	0.150	0.770	*
26	B	0.212	0.198	0.119	0.662	*
	C	0.189	0.148	0.139	0.737	*
	D	0.189	0.159	0.142	0.752	*
17	B	0.269	0.213	0.248	0.921	*
	C	0.299	0.215	0.284	0.921	*
	D	0.241	0.174	0.253	1.049	*
27	B	0.244	0.213	0.173	0.709	*
	C	0.223	0.176	0.190	0.854	*
	D	0.198	0.166	0.160	0.758	*
*Lower arch*						
33	B	0.222	0.177	0.219	0.989	*
	C	0.212	0.166	0.160	0.758	*
	D	0.198	0.148	0.154	0.778	*
43	B	0.208	0.168	0.140	0.672	*
	C	0.181	0.136	0.147	0.809	*
	D	0.172	0.136	0.130	0.757	*
34	B	0.189	0.167	0.101	1.572	*
	C	0.189	0.167	0.106	0.535	*
	D	0.180	0.159	0.113	0.627	*
44	B	0.197	0.177	0.128	0.647	*
	C	0.164	0.139	0.123	0.754	*
	D	0.162	0.134	0.115	0.712	*
35	B	0.216	0.191	0.138	0.640	*
	C	0.179	0.152	0.131	0.729	*
	D	0.181	0.155	0.121	0.669	*
45	B	0.209	0.195	0.111	0.531	*
	C	0.186	0.155	0.144	0.774	*
	D	0.174	0.159	0.103	0.591	*
36	B	0.236	0.189	0.169	0.716	*
	C	0.225	0.189	0.151	0.671	*
	D	0.205	0.154	0.142	0.695	*
46	B	0.210	0.189	0.120	0.573	*
	C	0.246	0.198	0.203	0.825	*
	D	0.185	0.162	0.141	0.762	*
37	B	0.290	0.220	0.275	0.948	*
	C	0.335	0.255	0.277	0.829	*
	D	0.295	0.220	0.246	0.834	*
47	B	0.262	0.223	0.168	0.644	*
	C	0.288	0.207	0.268	0.929	*
	D	0.194	0.162	0.144	0.740	*

SD: standard deviation

Cv: variation coefficient

*p < .0001

Finally, by calculating the difference in the values referred to both the linear measurements (Table 5) and the inter-arch distances (Table 6) between the units Carestream—CEREC, Carestream—Trios and CEREC—Trios, however much they differ from the impressions in PVS and showed no statistically significant differences (*p* < 0.05) between the different intraoral scanners. *Post hoc* analysis shows the combinations between the units, looking for those differences they have total significance determined, revealing the significance of each tooth between unit A and units B, C and D (Table 7). In particular, as regards the single elements, the Trios presented the trend of greater precision.

**Table 5:** Comparison between units B (Carestream CS 3600), C (CEREC Omnicam) and D (Trios 3Shape) for linear measurements (mm)

	B–C	B–D	C–D
	Estimate	Estimate	Estimate
*Upper arch*			
13	−0.023	−0.032	−0.009
23	−0.006	−0.008	−0.002
14	0.001	−0.020	−0.021
24	−0.026	−0.025	0.001
15	−0.039	−0.036	0.003
25	−0.014	−0.013	0.001
16	−0.003	−0.020	−0.017
26	−0.023	−0.022	0.001
17	0.030	−0.027	−0.057
27	−0.021	−0.046	−0.025
*Lower arch*			
33	−0.010	−0.024	−0.014
43	−0.026	−0.035	−0.009
34	−0.018	−0.009	0.009
44	−0.034	−0.036	−0.002
35	−0.036	−0.035	0.001
45	−0.023	−0.036	−0.013
36	−0.011	−0.031	−0.02
46	0.036	−0.025	−0.061
37	0.045	0.005	−0.04
47	0.027	−0.068	−0.095

**Table 6:** Comparison between units B (Carestream CS 3600), C (CEREC Omnicam) and D (Trios 3Shape) for inter-arch distances (mm)

	B–C	B–D	C–D
	Estimate	Estimate	Estimate
*Upper arch*			
23–13	−0.028	−0.053	−0.025
24–14	0.033	0.008	−0.025
25–15	0.067	0.031	−0.037
26–16	0.085	0.061	−0.025
27–17	0.180	0.109	−0.071
*Lower arch*			
43–33	−0.066	−0.084	−0.018
44–34	−0.004	−0.067	−0.064
45–35	−0.032	0.006	0.038
46–36	0.160	0.094	−0.066
47–37	0.125	−0.001	−0.126

**Table 7:** *Post hoc* analysis regarding the statistically significant differences between the units per tooth

	Contrast	Estimate	Asymptotic LCL	Asymptotic UCL	p-value
*Upper arch*					
13	A–B	−0.186	−0.225	−0.147	0.000
	A–C	−0.163	−0.202	−0.124	0.000
	A–D	−0.154	−0.194	−0.115	0.000
23	A–B	−0.153	−0.192	−0.114	0.000
	A–C	−0.147	−0.186	−0.108	0.000
	A–D	−0.145	−0.184	−0.106	0.000
14	A–B	−0.154	−0.191	−0.118	0.000
	A–C	−0.155	−0.192	−0.119	0.000
	A–D	−0.134	−0.170	−0.098	0.000
24	A–B	−0.174	−0.210	−0.138	0.000
	A–C	−0.148	−0.184	−0.111	0.000
	A–D	−0.149	−0.185	−0.112	0.000
15	A–B	−0.182	−0.218	−0.145	0.000
	A–C	−0.143	−0.180	−0.107	0.000
	A–D	−0.146	−0.182	−0.109	0.000
	B–C	0.039	0.002	0.075	0.032
25	A–B	−0.161	−0.198	−0.125	0.000
	A–C	−0.147	−0.183	−0.110	0.000
	A–D	−0.148	−0.185	−0.112	0.000
16	A–B	−0.194	−0.231	−0.158	0.000
	A–C	−0.191	−0.227	−0.154	0.000
	A–D	−0.174	−0.211	−0.138	0.000
26	A–B	−0.190	−0.227	−0.154	0.000
	A–C	−0.167	−0.204	−0.131	0.000
	A–D	−0.168	−0.204	−0.131	0.000
17	A–B	−0.242	−0.279	−0.206	0.000
	A–C	−0.272	−0.309	−0.236	0.000
	A–D	−0.215	−0.251	−0.178	0.000
	C–D	0.058	0.021	0.094	0.000
27	A–B	−0.223	−0.260	−0.187	0.000
	A–C	−0.202	−0.238	−0.165	0.000
	A–D	−0.177	−0.214	−0.141	0.000
	B–D	0.046	0.010	0.082	0.006
*Lower arch*					
33	A–B	−0.162	−0.201	−0.123	0.000
	A–C	−0.152	−0.191	−0.112	0.000
	A–D	−0.138	−.0177	−0.098	0.000
43	A–B	−0.139	−0.179	−0.100	0.000
	A–C	−0.113	−0.152	−0.073	0.000
	A–D	−0.104	−0.143	−0.065	0.000
34	A–B	−0.130	−0.167	−0.094	0.000
	A–C	−0.112	−0.149	−0.076	0.000
	A–D	−0.121	−0.158	−0.085	0.000
44	A–B	−0.144	−0.180	−0.107	0.000
	A–C	−0.110	−0.146	−0.073	0.000
	A–D	−0.108	−0.144	−0.071	0.000
35	A–B	−0.154	−0.191	−0.118	0.000
	A–C	−0.118	−0.154	−0.081	0.000
	A–D	−0.119	−0.156	−0.083	0.000
45	A–B	−0.153	−0.189	−0.116	0.000
	A–C	−0.130	−0.166	−0.093	0.000
	A–D	−0.117	−0.153	−0.081	0.000
36	A–B	−0.183	−0.220	−0.147	0.000
	A–C	−0.172	−0.208	−0.135	0.000
	A–D	−0.152	−0.188	−0.115	0.000
46	A–B	−0.149	−0.186	−0.113	0.000
	A–C	−0.185	−0.221	−0.148	0.000
	A–D	−0.124	−0.160	−0.087	0.000
	C–D	0.061	0.025	0.098	0.000
37	A–B	−0.236	−0.273	−0.200	0.000
	A–C	−0.281	−0.317	−0.244	0.000
	A–D	−0.241	−0.278	−0.205	0.000
	B–C	−0.044	−0.081	−0.008	0.010
	C–D	0.040	0.003	0.076	0.029
47	A–B	−0.205	−0.242	−0.168	0.000
	A–C	−0.232	−0.269	−0.195	0.000
	A–D	−0.137	−0.173	−0.100	0.000
	B–D	0.068	0.032	0.104	0.000
	C–D	0.095	0.058	0.131	0.000

A: PVS impression; B: Carestream CS 3600; C: CEREC Omnicam; D: Trios 3Shape

*p* < .05

**Fig. 2:**
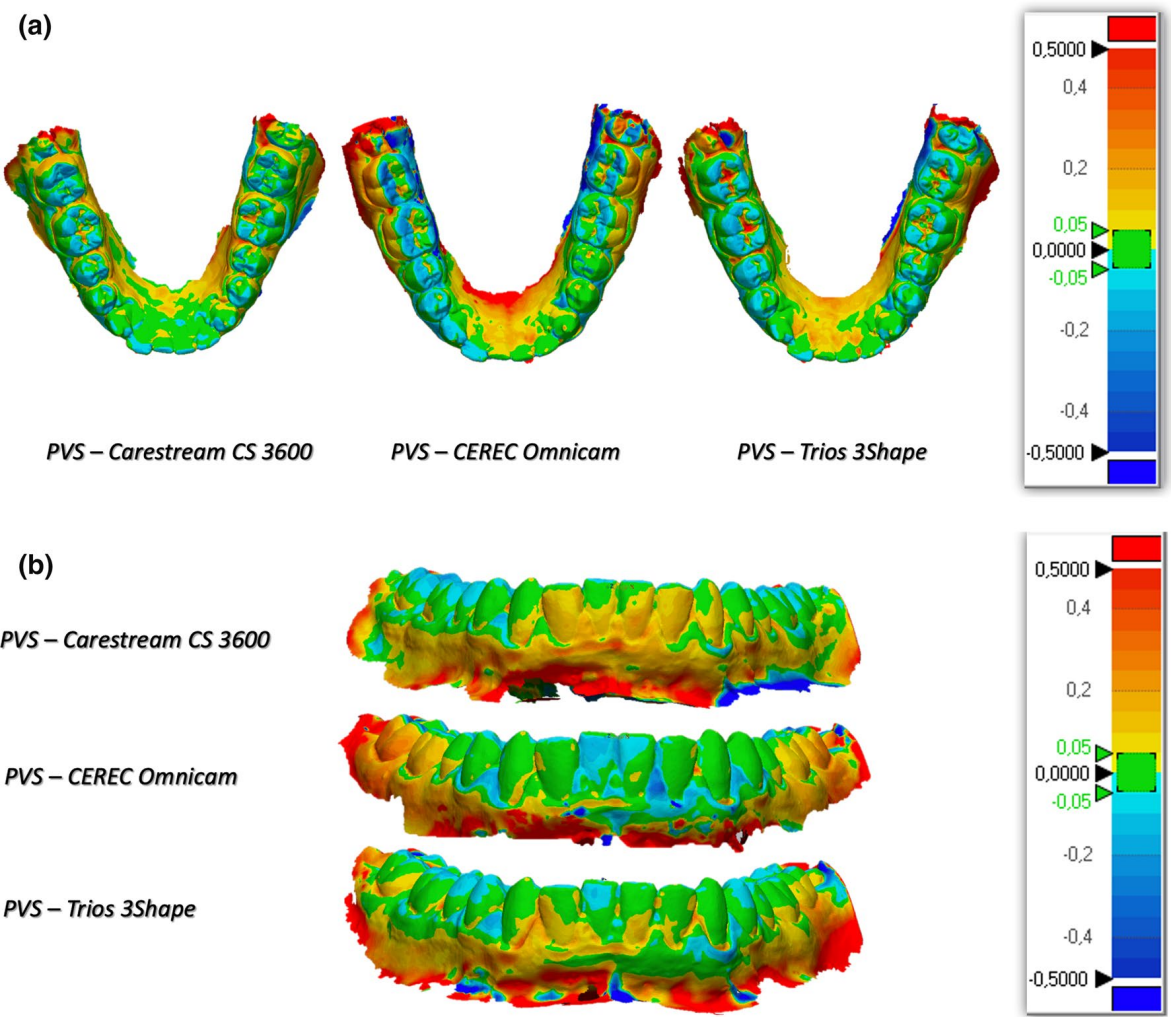
**a** Occlusal view of the 3D comparison of units B (Carestream CS 3600), C (CEREC Omnicam) and D (Trios 3Shape) with the PVS impression (A). **b** Frontal view of the 3D comparison of units B (Carestream CS 3600), C (CEREC Omnicam) and D (Trios 3Shape) with the PVS impression (A)

The 3D comparison showed that the scans deriving from CEREC more often show the blue areas, indicating an under-dimension of the measured data, while the comparisons with the Carestream show different protrusions. The areas of greatest discrepancy both in excess and in defect with respect to the PVS impression are the molar areas and incisal margins (Fig. 2a, b).

**Fig. 3:**
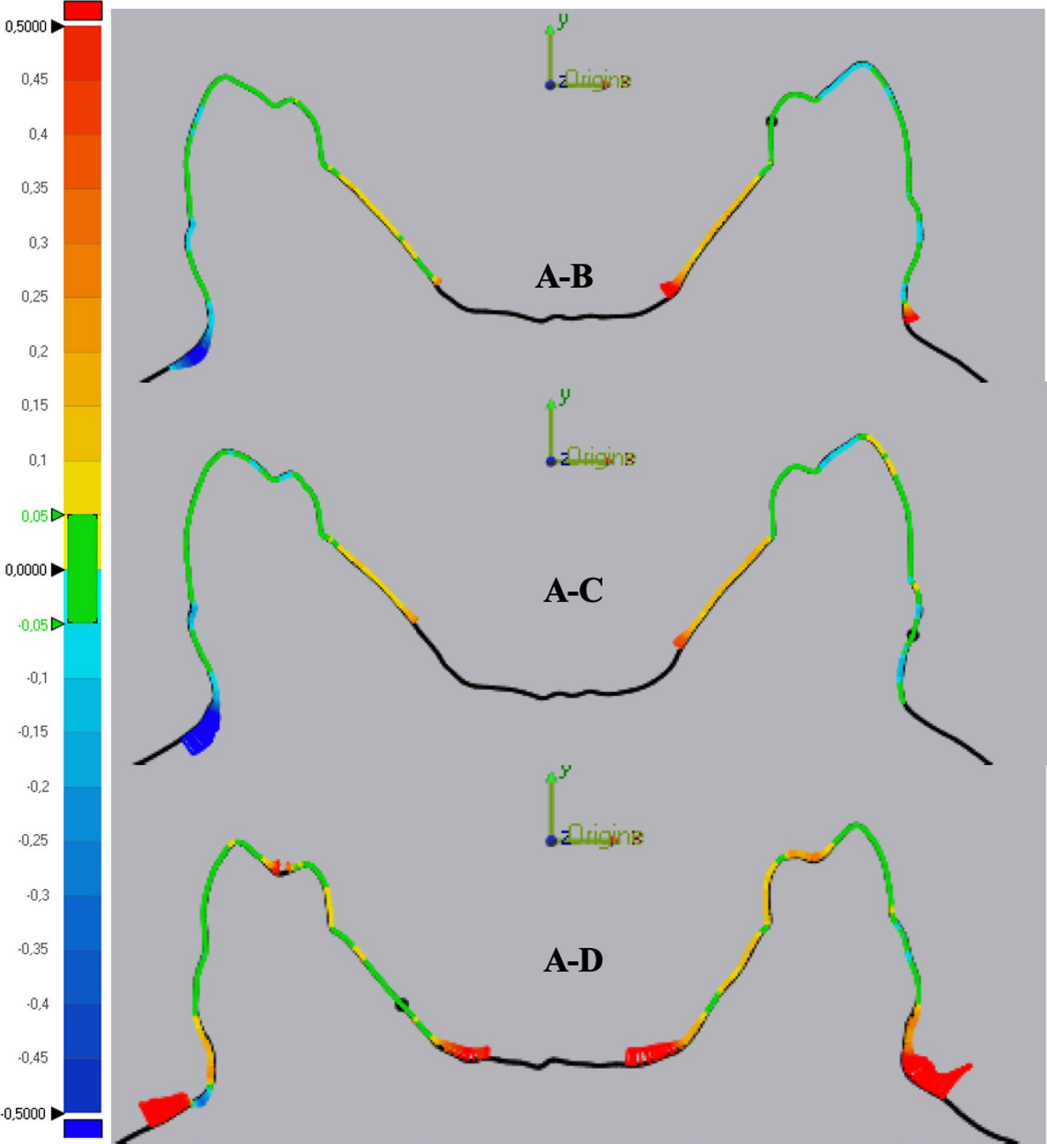
Cross section of elements 4.4–3.4 comparing units **A** (PVS impression), **B** (Carestream CS 3600), **C** (CEREC Omnicam) and **D** (Trios 3Shape)

The same trends resulting from the statistical analysis of the data and the 3D comparison are highlighted in the 2D analysis of the inter-arch cross sections for each element analyzed. Unit CEREC demonstrates a prevalence of light blue/blue color on the occlusal surfaces of the elements, while in the cross section 4.4–3.4 of unit Trios yellow/red occlusal surfaces are observed which confirm the average variation of the difference in inter-arch distance in the comparison between units A - D approximately 60 µm greater than the A - B differences (Fig. 3).

As for the acquisition times, the scanner that recorded the shortest times (6 min on average) and therefore with a more performing speed was the Trios, but the one that had the most uniform behavior in terms of performance was the Carestream (Table 8).

**Table 8:** Mean caption time (minutes)

	Mean	Std
A	13	2
B	10	2
C	14	5
D	10	4

## Discussion

The introduction on the market of intraoral scanners (IOS) allows a direct scan of the dental arches, from which a virtual or physical 3D model can be obtained, thanks to three-dimensional printing. The digital file can be stored and used without limits, representing an optimal solution in conditions in which it is necessary to remake, for example, an orthodontic setup or a prosthetic element [22].

The main feature to be evaluated in an intraoral scanner is accuracy, the result of the analysis of trueness and precision [4, 23–27], which mainly depend on the scanner's acquisition / processing software. In the literature, the conventional impression is used as a comparison measure to evaluate the accuracy of intraoral scans, in particular polyvinylsiloxane as it is the most accurate material among those on the market [28].

All the IOS systems analyzed showed a similar distortion pattern, with the worst trueness values ​​in the category of linear measurements, with a general trend of greater deviation in the molar area. Considering the level of accuracy on the single tooth, it can be said that the Trios 3Shape scanner was found to be the closest to the “real”. In accordance with the results obtained from the present study, in the literature it has been shown that there is an evident distortion pattern in the optical impression in correspondence with the posterior region of both dental arches [2, 29–31], while the anterior zone expresses greater accuracy [2]. Increasing the scan area increases the *merging* and consequently a possible error. Optical technology is based on the emission of light at the surface of the dental element and the capture of the reflected ray: an excessive reflection phenomenon due to, for example, metal reconstructions, saliva, crowding or difficult access areas, can affect the quality and sharpness of captured images. This can lead to a progressive distortion of the impression greater than 100 µm and consequently to a reduction in trueness, particularly in the molar region [29]. Moreover, any slight movement of the hand during the scanning process contributes to the error in the collection of digital data.

From the 3D analysis of the impressions, the present in vivo study found deviations with negative values in the molar area for CEREC. The error in the anterior sextant could be due to the overlapping of the partial scans referring to the two hemi-arches and the morphology of the elements, in fact from the 3D comparison the deviation at the level of the incisal margins was evident. Malik et al. [32] performed a 3D comparison between PVS impression and two optical impressions scanned with CEREC and Trios of a full arch reference model from which it is concluded that despite the statistical significance of the differences found between conventional and digital, the deviation in terms of trueness and precision, however, remains within the ± 100 µm range for both scanners. This study reports statistically significant and similar values: the deviation from the PVS impression was greater in the molar area.

By comparing the intraoral scanners between them, it can be stated that there are no statistically significant differences between one and the other; this has been reported by this in vivo study and is confirmed by the literature. The study by Winkler et al.[33] compares Trios and Carestream about accuracy over the entire upper dental arch. The difference in accuracy between the two scanners was approximately 10 μm, with the Trios superior to the Carestream, but not clinically significant. However, in some cases of severe dental crowding, they can be less precise. Indeed, other studies in the literature have reported significant differences between scanners, in particular high accuracy values for Trios 3Shape and 3 M True Definition scanner compared to CEREC Omnicam [34, 35].

It is important to underline that a limitation of the in vitro studies seen so far is represented by the different optical properties of the materials used for the creation of the reference models (resins, gums, metal, gypsum) compared to the properties of intraoral structures: natural teeth have various degrees of translucency and refraction, as well as the materials used for restorations and the soft tissues of the oral cavity [36].

Moreover, the scan acquisition time was compared with the time required to obtain the conventional impression. In the study by Grünheid et al.[26], considering the time required to produce the alginate and that of software improvement, there are no statistically significant differences between the two techniques. On the contrary, in this in vivo study it was deduced that Trios and Carestream are faster than the PVS impression, while CEREC was not more performing than silicone, but this may be related to the computers or to the software version of the program.

Considering the progressive and dynamic updating of digital systems and optical technologies, further in vivo studies are needed to carefully evaluate the progress of these technologies in the dental field and compare the results obtained from in vitro studies with the performance of IOS in vivo, considering all possible variables which can alter the qualities of the intraoral scanner. Clinical practice will guide the dentist toward the choice of a certain intraoral scanner rather than another. For example, this in vivo study showed that the Trios 3Shape is more accurate on the single dental element than the transverse dimension of the complete arch, so a prosthetist might prefer this scanner for partial fixed prosthetic reconstructions. On the other hand, the Carestream CS 3600 showed a better performance on the interarch diameters, expression of a greater approach to the real dimension of the complete arch. This may be more in line with the needs of an orthodontist, who typically works on whole arches.

## Conclusions

From the present in vivo study, it follows that the null hypothesis was rejected:

- The Trios 3Shape was found to be the most accurate single-tooth scanner, while the Carestream CS 3600 showed better inter-arch diameter performance compared to PVS impressions.
- Despite this, there is no statistically significant difference between the three IOS systems in the impressions accuracy analysis of complete dental arches.
- The 3D and 2D analyses showed a trend of greater distortion of the impressions compared to the conventional one in the molar region. Measurements on single points and inter-arch distances confirm this trend.
- Carestream CS 3600 and Trios 3Shape recorded a shorter mean scan time compared to the CEREC Omnicam and the silicone impression.

## Data Availability

The datasets used and/or analyzed during the current study are available from the corresponding author on reasonable request.
